# Optimizing Culture Medium Composition to Improve Oligodendrocyte Progenitor Cell Yields *In Vitro* from Subventricular Zone-Derived Neural Progenitor Cell Neurospheres

**DOI:** 10.1371/journal.pone.0121774

**Published:** 2015-04-02

**Authors:** Paula G. Franco, Juana M. Pasquini, Lucas Silvestroff

**Affiliations:** Departamento de Química Biológica, Facultad de Farmacia y Bioquímica, Universidad de Buenos Aires, and Instituto de Química y Fisicoquímica Biológicas “Profesor Alejandro C. Paladini” (IQUIFIB), UBA-CONICET, Ciudad Autónoma de Buenos Aires, Argentina; Hospital Nacional de Parapléjicos – SESCAM, SPAIN

## Abstract

Neural Stem and Progenitor Cells (NSC/NPC) are gathering tangible recognition for their uses in cell therapy and cell replacement therapies for human disease, as well as a model system to continue research on overall neural developmental processes *in vitro*. The Subventricular Zone is one of the largest NSC/NPC niches in the developing mammalian Central Nervous System, and persists through to adulthood. Oligodendrocyte progenitor cell (OPC) enriched cultures are usefull tools for *in vitro* studies as well as for cell replacement therapies for treating demyelination diseases. We used Subventricular Zone-derived NSC/NPC primary cultures from newborn mice and compared the effects of different growth factor combinations on cell proliferation and OPC yield. The Platelet Derived Growth Factor-AA and BB homodimers had a positive and significant impact on OPC generation. Furthermore, heparin addition to the culture media contributed to further increase overall culture yields. The OPC generated by this protocol were able to mature into Myelin Basic Protein-expressing cells and to interact with neurons in an *in vitro* co-culture system. As a whole, we describe an optimized *in vitro* method for increasing OPC.

## INTRODUCTION

Cell transplantation therapy is a promising strategy for neurodegenerative diseases, where newborn brain progenitors seem to be abundant and malleable sources of neural cells. Particularly, optimizing oligodendrocyte progenitor cell (OPC) cultures is a vital prerequisite for successful cell replacement therapy strategies when treating demyelinating disorders (reviewed in Grade et al., 2013) [1] or for *in vitro* purposes.

One of the original methods for OPC isolation was published by McCarthy and de Vellis (1980) [2] and stands out for being economic. However, OPC proliferation is inhibited in high densitiy cultures [14]. Variations of this culture method include supplementation of media with specific growth factors such as Platelet derived Growth factor-AA (PDGF-AA) [4] or B104 conditioned medium [5]. Immunopanning techniques [6, 7] are able to increase OPC purity at the expense of a low yield. Immunomagnetic cell sorting is an alternate strategy [8, 9] that uses less antibodies than immunopanning, although does not solve the low OPC yield obstacle.

We have based our study design to increase OPC proportions in an *in vitro* cell culture system by modifying the culture media components. Since Platelet-Derived Growth Factor Receptor alpha (PDGFRα) is expressed by OPC, and PDGFRα^+^ cells are the main source of myelinating cells in human and mice Central Nervous System (CNS) [10, 11], we targeted this signaling pathway to selectively amplify OPC populations *in vitro* from newborn mouse subventricular zone (SVZ)-derived neurosphere (NS) cultures.

The PDGF protein family plays a crucial role in the CNS as from early development [12], throughout adulthood and during disease. It has been documented that astrocytes and neurons physiologically synthesize and secrete PDGF, and also express PDGFR [13, 14] while OPC only express the PDGFR [15]. In addition, Moore et al. (2014) [16] have described SVZ progenitors expressing both PDGFR α and β genes.

Among many roles, PDGF are known to regulate cell proliferation by activating the PDGFR intracellular Tyrosine Kinase Domain through several pathways [17]. In addition to OPC proliferation, PDGF signaling has also been linked to neural stem cell (NSC) commitment to the oligodendroglial lineage [18], similar to that described for mesenchymal stem cells multipotency restriction [19]. The PDGF-AB heterodimer has been described to regulate OPC proliferation *in vitro* [20] and SVZ-derived oligodendrogenesis *in vivo* [21]. PDGF-AA has been used *in vivo* to replenish endogenous OPC in experimental CNS demyelination models [22], although it has been known to participate in glioma formation [23]. Nonetheless, PDGF-AA has been widely used to expand OPC *in vitro* from pluripotent stem cells [18] and NSC [24]. The B104 neuroblastoma cell conditioned media has been used as an alternate source of PDGF-AA for *in vitro* approaches as well [25, 26, 27].

Although less popular, PDGF-BB is not a foreign molecule to the CNS, since it is synthesized by embryonic cortical NSC and neural progenitor cells (NPC) *in vitro* [28]. PDGF-BB null mice generate litter that die shortly after birth [3], while its over-expression *in vivo* is sufficient to drive cell proliferation and generate CNS gliomas enriched in NG2^+^/GFAP^-^ cells [29]. Chojnacki and Weiss (2004) [30] indicate that PDGF-AA and BB homodimer-responsive progenitors are present in the CNS as from early prenatal stages of development in the medial embryonic eminences, one of the brain structures preceding the postnatal ventricular and subventricular zone brain structures.

In the persuit of increasing OPC proportions *in vitro*, the basic Fibroblast Growth Factor (bFGF) played a key role in our experimental design. bFGF positively regulates oligodendrogenesis from spinal cord and telencephalon progenitor cell cultures [31, 32]. Oligodendrogenesis from embryonic ventricular zone progenitors *in vivo* is increased in response to bFGF as well [33, 34], and is mediated by the FGF receptors 1 and 2 [35]. bFGF also favours the proliferation of OPC isolated from whole brain and corpus callosum tissues [20, 36], and increases the expression of PDGFRα on OPC, making them all the more sensitive to PDGF extracellular ligands [37]. In addition, the proliferation of SVZ-derived and striatal neurosphere-derived OPC is augmented by bFGF [38, 21].

Since our interest is focused in developing highly pure OPC cultures, we evaluated and compared the effects of both PDGF-AA and PDGF-BB treatment on OPC generation from postnatal SVZ-derived primary NS cultures. As reported by Kelly et al. (1991) [39] PDGF-BB binds to human PDGFRα with a lower affinity than PDGF-AA, however it is considered to be of high affinity at PDGF-BB physiological concentrations.

Heparin, which has been shown to increase adult mouse NS overall culture yields [40], was also used to improve culture efficiency. As a whole, combining Heparin with bFGF/PDGF-BB had the best result in terms of the final OPC proportions and overall OPC yield.

Even though adding a PDGF-BB and bFGF combination during NS formation reduced the overall cell biomass, it effectively favored the highest OPC proportions.

In this report, we describe a protocol for generating high yield OPC-enriched cultures by supplementing culture medium with bFGF and PDGF-BB which could be used for cell grafting source as well as an *in vitro* model to further understand the biology of oligodendrocyte differentiation and remyelination processes.

## MATERIALS AND METHODS

### Materials

The cell culture plastic ware was from Greiner BioOne. The DMEM/F12 culture media, B27 Supplement (without antioxidants) and L-Glutamine were from Life Technologies. Poly-L-Lysine (Mol. Weight ≥ 300.000) and Triiodo Thyronine were from Sigma. The human PDGF-AA, human IGF-1 and human EGF were all recombinant and from Peprotech (cat. # 100–13A, 100–11 and AF-100–15, respectively). The recombinant human bFGF was a gift from Dr. Baldi (IBYME, Argentina) and the recombinant human PDGF-BB was a generous gift from Laboratorios Beta S.A. (Argentina). Fetal Calf Serum was purchased from Natocor (Argentina) and Heparin was from Nortia S.A. (Argentina). Antibodies; anti NG2 (Chemicon), anti PDGFRα (Neuromics), anti MBP (gift from former Campagnoni Lab, UCLA, USA), anti GFAP (Neuromics) and anti βTubulin III (Sigma). Further details on antibodies are listed in Table 1. The peptides sequences of PDGF proteins were the following:

PDGF-AA variant 1:

SIEEAVPAVC KTRTVIYEIP RSQVDPTSAN FLIWPPCVEV KRCTGCCNTS SVKCQPSRVH HRSVKVAKVE YVRKKPKLKE VQVRLEEHLE CACATTSLNP DYREEDTGRP RESGKKRKRK RLKPT.

PDGF-BB:

SLGSLTIAEP AMIAECKTRT EVFEISRRLI DRTNANFLVW PPCVEVQRCS GCCNNRNVQC RPTQVQLRPV QVRKIEIVRK KPIFKKATVT LEDHLACKCE TVAAARPVT.

**Table 1:** Descriptive information of antibodies used for Immunocytochemistry.

Antibody Name	Clonality	Host Species	Supplier	Catalogue #	Clone ID	Immunogen*	Final Dilution
βTubulin III	Monoclonal	Mouse	Sigma Aldrich	T8578	2G10	Synthetic peptide corresponding to amino acids 436–450 of neuronal specific βIII Tubulin.	1:200
GFAP	Polyclonal	Chicken	Neuromics	CH22102	-	Purified bovine spinal cord	1:100
MBP	Polyclonal	Rabbit	Kind gift from the fromer Campagnoni Lab†.	-	-	N-terminal fragment of rat MBP (amino acids 1–154).	1:300
NG2	Polyclonal	Rabbit	Millipore	AB5320	-	Immunoaffinity purified NG2 Chondroitin Sulphate Proteoglycan from rat.	1:200
Olig2	Monoclonal	Mouse	Millipore	MABN50	211F1.1	Recombinant protein corresponding to human Olig2	1:100
PDGFRα	Polyclonal	Goat	Neuromics	GT15150	-	NS0-derived, recombinant mouse plateletderived growth factor receptor alpha (rmPDGFR) extracellular domain.	1:100

*Antigen used to raise the antibodies.

†UCLA, USA.

### Animals

The CNP::EGFP transgenic mice expressing Enhanced Green Fluorescent Protein (EGFP) under the 2',3'-Cyclic-nucleotide 3'-phosphodiesterase (CNP) promoter have been previously characterized [41, 42], while the Actin::EGFP (Act::EGFP) mice express the EGFP reporter under Actin gene promoter. Both transgenic and wild type mice used in the present work had a C57BL/6J genetic brackground. All techniques involving animals for this study were performed in agreement with the Buenos Aires University School of Pharmacy and Biochemistry Guidelines for Laboratory Animal Welfare Committee (*Comité de ética para el uso de animales de laboratorio de la Facultad de Farmacia y Bioquímica—UBA*). The perinatal mice (2 to 4 days of age) were euthanized by decapitation as a means of obtaining undamaged SVZ brain tissue for our studies that was free from anaesthetic chemical contaminants. The documentation indicating the *Comité de ética para el uso de animales de laboratorio de la Facultad de Farmacia y Bioquímica—UBA* specifically approved our experimental protocols can be found in the Buenos Aires University School of Pharmacy and Biochemistry Resolution N° 3228/14.

### NS Cell Culture

For NS cultures, the SVZ was isolated from mice pups between postnatal day 2 and 4. The brains were removed from mice skulls under aseptic conditions. The SVZ tissue isolation itself was performed similar to the technique described by Mirzadeh et al. (2010) [43]. Briefly, we made an initial transversal cut across the whole brain, and then hemispheres were separated through the longitudinal mid-line. The hippocampus was then removed from the rostral brain portions to access the lateral ventricles. The isolated SVZ tissue of a single animal was mechanically dissociated with a p1000 automatic Gilson pipette in 3,5 cm (Ø) plastic petri dishes containing 1 ml of DMEM/F12. The suspension was added to a capped conical tube containing 9 ml of DMEM/F12, and centrifuged for 5 min at 300 xg at room temperture. The supernatant was discarded and the cell pellet was resuspended in 2% B27-supplemented DMEM/F12. Growth factors (bFGF, EGF, PDGF-AA, PDGF-BB) were then added at a final concentration of 20 ng/ml and in different combinations. In some experiments we also added Heparin at varying concentrations during the proliferation stage of cell culture.

### NS radius size

The NS were photographed while suspended in culture media, where more than one representative field image was taken for each well, plate or flask. The two dimensional images were analyzed with the Image ProPlus Software by measuring the NS radius and data was compared among the different treatments.

### NS dissociation and plating

For Immunocytochemistry (ICC) analysis, NS were mechanically dissociated and cells were seeded on poly-L-Lysine coated coverslips and cultured for 2 days. When required, we triggered cell differentiation by culturing cells for 6 aditional days in DMEM-F12/B27 in the absence of growth factors, and in some cases, with the addition of T3, IGF-1 or FCS (48 ng/ml, 20 ng/ml and 1% final concentration, respectively).

### Immunocytochemistry (ICC)

The cells attached to poly-L-Lysine coated glass coverslips were rinsed once with 0.1 M Phosphate Buffered Saline (PBS; 8 g/L NaCl, 0.2 g/L KCl, 0.24 g/L KH_2_PO_4_, 1.44 g/L Na_2_HPO_4_, pH 7.4) at room temperature. Then they were fixed in 4% paraformaldehyde for 30 minutes. Cells were rinsed with PBS and blocked in 5% FCS (diluted in PBS) overnight at 4°C. Cells were directly incubated with antibodies in a humidified chamber. All antibodies were diluted in a 1% FCS – 0.1% Triton X-100 – PBS solution and incubated overnight at 4°C. Coverslips were rinsed with PBS after primary and secondary antibody incubations. Höechst H33258 dye was added to the secondary antibody solution to a final concentration of 5 μg/ml to stain cell nuclei. Coverslips were mounted onto glass slides with a Mowiol 4–88 mounting solution. Anti PDGFRα and NG2 antibodies were used as markers for oligodendroglial progenitor cells. MBP antibody detects mature oligodendrocytes, GFAP was used to label astrocytes, βTubulin III for neurons and Olig2 was used for progenitor cells.

### Fluorometry and protein quantitation

At the end of the proliferation stage NS were recovered from multiwell plates and placed in 1.5 ml capped microtubes. NS were centrifuged 2 min at 10.000 rpms in a MiniSpin Plus Eppendorf bench table centrifuge at room temperature. The supernatant was discarded. The cells were rinsed once in PBS and the pellet was frozen at -20°C until use. For Höechst and EGFP fluorescence multiplex analysis, the frozen cell pellet was thawed and resuspended in 50 μl of TNE buffer (10 mM Tris base, 0.2 M NaCl, 1 mM EDTA) containing Höechst H 33258 (5 μg/ml), according to Gallagher (2000) [44]. The resuspended whole cell homogenate was placed in Cell Star (Grenier BioONE) white 96-well plates and analyzed with an LS 55 Fluorescence Spectrometer. Both DNA-bound Höechst and EGFP fluorescence were measured in each well. For total DNA analysis, excitation and emission wavelengths were set at 352 and 461 nm, respectively, according to the Höechst fluorescence spectra when bound to DNA. For EGFP fluorescence analysis, excitation and emission wavelengths were set at 488 and 509 nm, respectively. The same homogenate were then used to measure the protein content using the Bradford assay.

### Microscopy

The fluorescent microscopy image acquisition was performed with an Olympus BX50 fluorescence microscope. The image acquisition was performed with the CellSense software, while the image analysis was performed with the Image Pro Plus software. Red fluorescence in microscope images was converted to magenta. For quantitating cell proportions, immunopositive cells were counted from digital images and results were expressed normalized to total nuclei. For confocal imaging, we used an Olympus FV1000 microscope and images were processes with the FluoView software.

### Statistical analysis

All statistical analysis was performed with the GraphPad Prism software. Experiments were analyzed with Student´s t test, or One-Way ANOVA plus Student-Newman-Keuls or Dunnett´s post test. In cases where Two-Way ANOVA was applied, the analysis was accompanied by a Bonferroni post-test. Data regarding the statistical significances is indicated in the figure legends.

### Neuron and Neurosphere co-cultures

Neuron-enriched cultures were obtained from whole brains from embryonic day 14 WT mice with a protocol adapted from Kaech and Banker (2006) [45], Gardner et al. (2012) [46] and Xu et al. (2012) [47]. Briefly, brains from mice fetuses were isolated and dissociated with a scalpel to 1 mm size fragments. Then, tissue fragments were rinsed in PBS and digested with Trypsin at a 0.4% final concentration in 1% Glucose supplemented PBS for 10 minutes at 37°C. Large tissue fragments were allowed to spontaneously decant, while the upper homogenous cell suspension was isolated, rinsed in 9 ml DMEM/F12 and finally resuspended and plated in 2% B27-supplemented DMEM/F12 with Cytosine Arabinoside 2 μM final concentration. After 6 days, dissociated NS were plated on to the neuron enriched monolayer and co-cultured in DMEM/F12-B27 media for 6 additional days.

## RESULTS

### Improving OPC proportions in NS cultures

**Fig 1:**
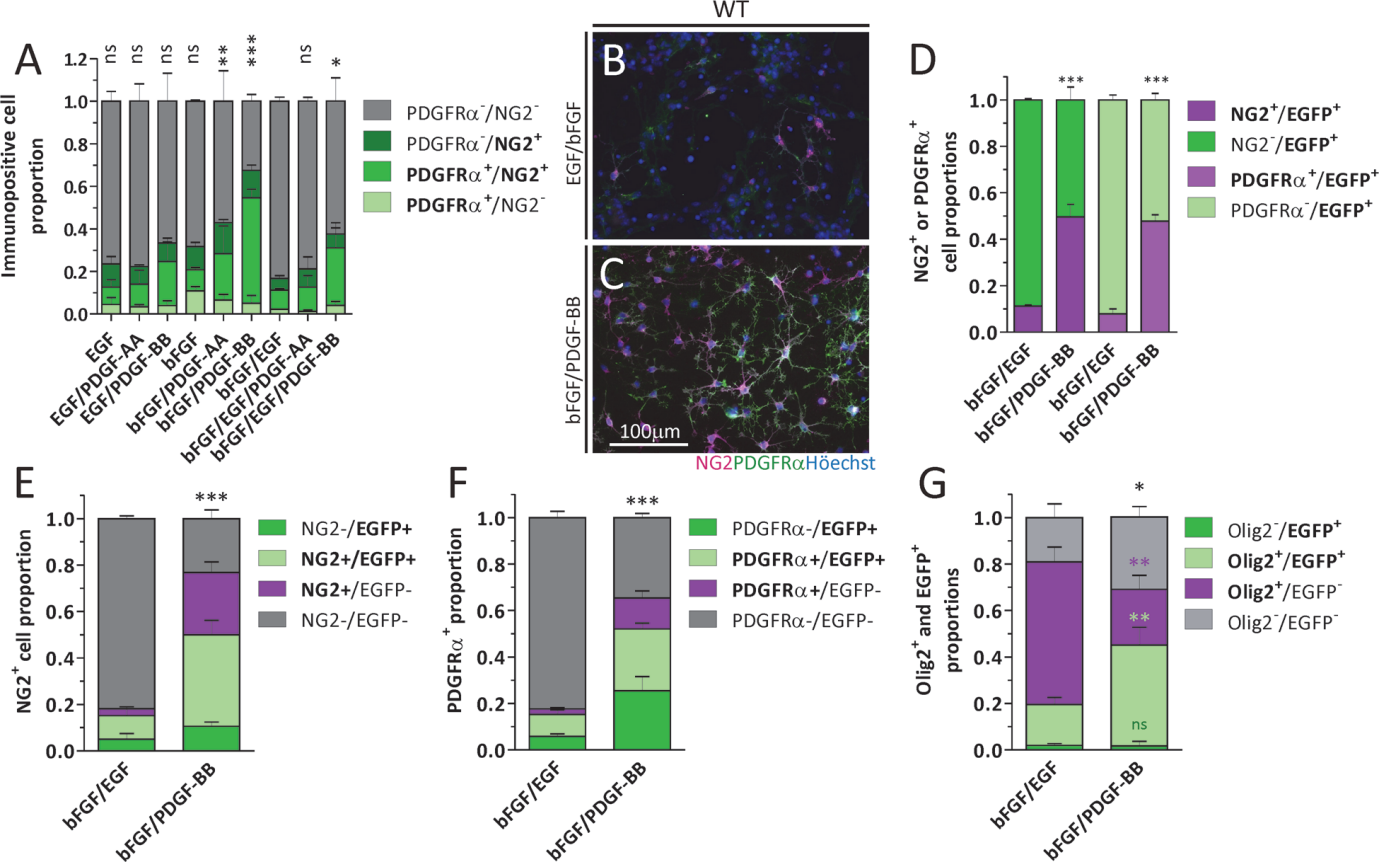
Detection of OPC markers by ICC on NS cells. A) Quantitation of NG2^+^ and/or PDGFRα^+^ cell proportions in WT mice NS generated in the presence of different growth factor combinations. Data belong to three independent experiments for each condition. Data for NG2^-^/PDGFRα^-^ was analyzed with a One-way ANOVA plus Dunnett´s post test, where bFGF/EGF was set as the control. B, C) Representative images of NG2^+^ and PDGFRα^+^ cells generated from WT mice cultures in the presence of either bFGF/EGF or bFGF/PDGF-BB. D) Comparison of NG2^+^ or PDGFRα^+^ cell proportions in NS cultures generated from Act::EGFP mice in the presence of bFGF/EGF or bFGF/PDGF-BB. Data for NG2^+^ (dark magenta) or PDGFRα^+^ (light magenta) cells was analyzed separately with Student´s t test. E, F) NG2 and PDGFRα immunodetection in CNP::EGFP derived cultures. Gray bars in each graph were analyzed with Student´s t test. G) Olig2 expression in WT mice NS cultures. Asterisks are colour coded to indicate the pairs of bars compared and analyzed with Student´s t test. Cell proportions in A, and D-G are expressed as a fraction of the total cell nuclei counted for each condition. Error bars represent the SD for all bar graphs. Scale bar in C equals 100 μm for B and C. ns = not significant, * = p < 0.05, ** = p < 0.01 and *** = p < 0.001.

One of the main aims of our work was to evaluate the potential of NS cells to generate oligodendrocytes (OL) under different culture conditions. We therefore analyzed NG2^+^ polydendrocytes and PDGFRα expressing cells by ICC to quantitate OPC proportions as a starting point. Both these markers overlapped in a high degree in this culture system. The effect of adding the standard EGF/bFGF growth factor combination during 6 days to the NS culture media was compared with the impact exerted by each factor on its own or combined with the PDGF homodimers (AA or BB). As previously observed in rat-SVZ cultures, mice NS generated NG2^+^ and PDGFRα^+^ cell populations that did not entirely overlap. After culturing NS with EGF and bFGF together, less than 20% of the cells were immunopositive for NG2 and/or PDGFRα (Fig. 1A). The highest NG2^+^ and/or PDGFRα^+^ cell proportions (more than 60%) were found in the bFGF/PDGF-BB condition, and the amount of NG2^-^/PDGFRα^-^ cells was statistically different (p < 0,01) from the bFGF/EGF condition. PDGF-AA, or PDGF-BB, on its own generated non-profitable yields in terms of amount and size of NS to justify continuing with an ICC protocol, and were excluded from the analysis. Though PDGF-AA is frequently used when an OPC culture enrichment is desired, we found that OPC ratios were higher in the bFGF/PDGF-BB condition than with bFGF/PDGF-AA. The EGF/bFGF combination was used as a control (CTL) for the remaining experiments, given that it is used in most NS culture protocols published by others. The NG2^+^ and/or PDGFRα^+^ cells obtained after the CTL or bFGF/PDGF-BB treatments had a typical OPC morphology as observed by ICC (Fig. 1B, C).

We confirmed these results by generating NS cultures from the SVZ of Act::EGFP and analyzing the NG2^+^ and PDGFRα^+^ cell proportions in CTL and bFGF/PDGF-BB-treated cultures (Fig. 1D and S1 A, B Fig.). We further repeated the immunodetection of these OPC markers in CNP::EGFP transgenic mice, which express EGFP in the oligodendroglial lineage as from the OPC stage (S1 C-F Fig.). Analysis of NG2^+^ and PDGFRα^+^ cells in these mice indicated that the proportion of OPC derived from NS grown in the presence of bFGF/PDGF-BB was higher than in the CTL condition (Fig. 1E, F). The OPC generation efficiencies in the WT and CNP::EGFP mice were comparable.

In addition, we analyzed the proportion of GFAP^+^ and βTubulin III^+^ cells in some of the above conditions (S1 G Fig.). The cell proportion for both these two markers was not appreciably different despite the significant differences in OPC proportions.

### Olig2 expression is shared among OPC and GFAP^+^ cells

We intended to use Olig2 as an additional marker to detect OPC enrichment. In the CNP::EGFP mice-derived cultures we observed Olig2^+^ cells were decreased in the bFGF/PDGF-BB cultures (Fig. 1G and S1 H-O Fig.). We also analyzed Olig2 expression in GFAP^+^ cells in WT mice cultures suspecting Olig2 was being expressed early in immature cells before reaching their OPC stage. We observed that almost half the GFAP^+^ cells co-expressed Olig2 in the control condition and the amount of Olig2^+^/GFAP^+^ cells were significantly reduced in the bFGF/PDGF-BB condition (S2 Fig.). We therefore avoided Olig2 as an indicator of oligodendroglial commitment in this culture system.

### NS culture yields after different growth factor treatments

Even when the bFGF/PDGF-BB-treated NS cultures generated the highest OPC proportions, we noticed that NS were significantly smaller in size compared to CTL NS. We found a reduction in NS size for all the analyzed conditions respect to CTL in WT-derived cultures (S3 A, B Fig.). Since the NS radii length measurement does not necessarily represent total cell culture yields, we recurred to additional parameters to estimate total cell amounts in the cultures. For this purpose, we took advantage of the Act::EGFP transgenic mice, which express EGFP in all viable cells under the Actin gene promoter. We found the average NS size in each treatment was similar between Act::EGFP and WT NS cultures (S3 A Fig.), indicating there were no mice strain-specific effects on NS radii. The bright field-green fluorescence merged images confirmed EGFP is expressed in all the cells of Act::EGFP cultures (S3 C Fig.). Furthermore, the cytoplasmic EGFP fluorescence was lost in small-sized cells with a pyknotic nuclei, indicating EGFP expression is only sustained in viable cells (Data not shown).

**Fig 2:**
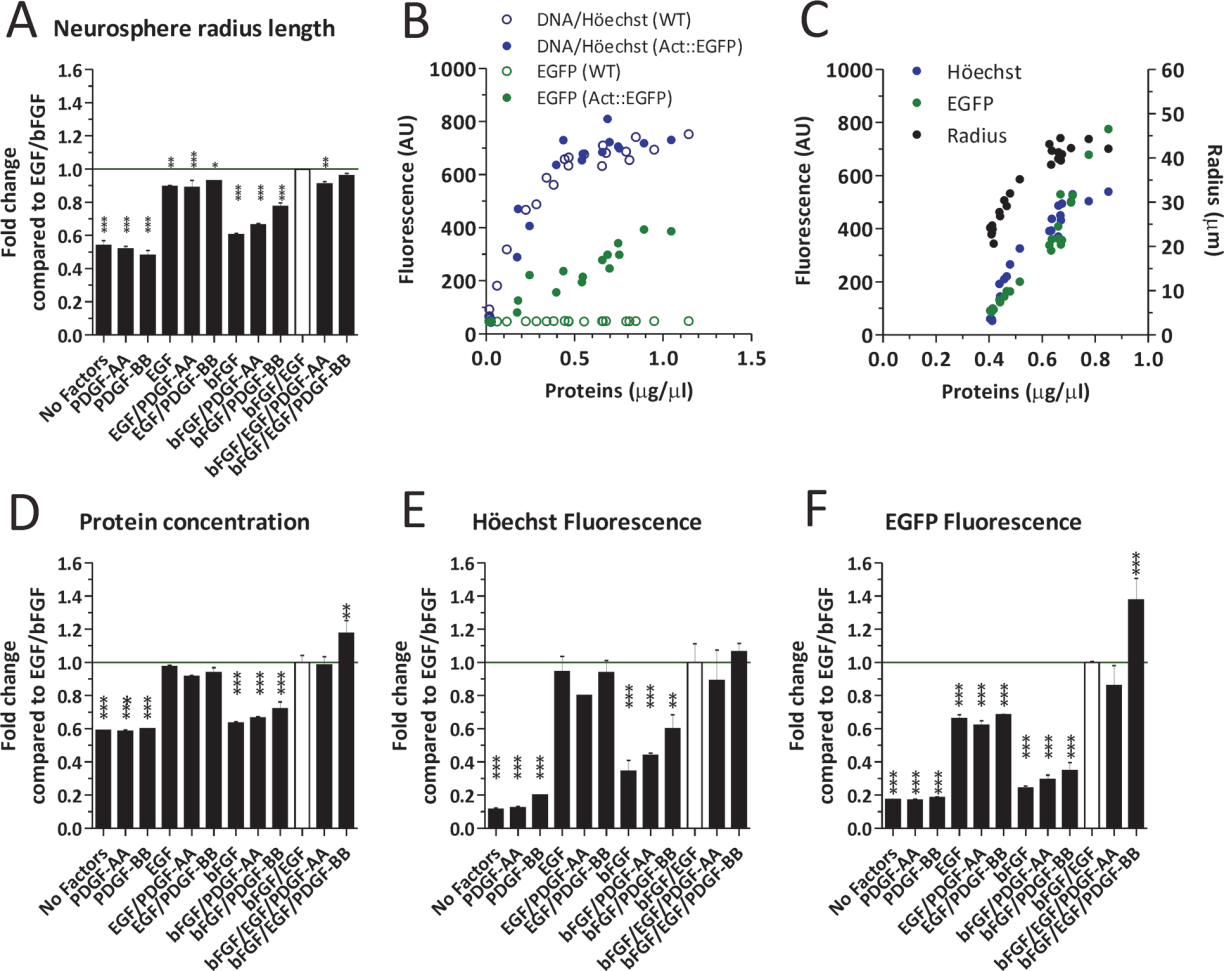
Analying the cell yield by measuring different parameters in Actin::EGFP (A-F) and WT (B) mice NS cultures. A) The NS radius length was measured under the different experimental conditions. B) Comparison of EGFP and Höechst fluorescence as a function of total proteins in NS cultures derived from WT and Actin::EGFP mice. C) The radius length, DNA content and EGFP fluorescence are expressed as a function of the protein concentration. D-F) Whole NS extracts were analyzed according to the total cell protein concentration (D), DNA content (E) and EGFP fluorescence (F) for different experimental conditions. Data in A, D-F were analyzed with a One-way ANOVA and Dunnet´s post-test, where the EGF/bFGF treatment was established as the control and error bars represent the SD. * = p < 0.05, ** = p < 0.01, *** = p < 0.001.

A multiple analysis of culture yields was performed by first measuring Act::EGFP NS radii after treatment with the different growth factors combinations. Adding bFGF on its own, or together with PDGF generates small sized NS compared to the CTL (Fig. 2A). There was a tendency to find larger NS when PDGF was added to the bFGF condition, being PDGF-BB more effective on increasing the NS size than PDGF-AA.

We therefore decided to reinforce the culture yield evaluation by measuring additional culture parameters. We first compared the EGFP fluorescence readout and DNA content between WT and Act::EGFP NS cultures (Fig. 2B). The DNA content increased as a function of the total protein concentrations equally in both WT and Act::EGFP mice. In addition, EGFP fluorescence only increased as a function of the total protein in the Act::EGFP mice cultures.

We found that the total protein content of Act::GFP NS extracts positively correlated with NS radius length, EGFP fluorescence of viable cells and the Höechst stained DNA (Fig. 2C). Furthermore, fluorescence corresponding to EGFP and DNA-bound Höechst also correlated with each other. We therefore used these alternative methods to evaluate the NS culture yields after treatment with each growth factor combination (Fig. 2D-F). Overall, the parameters analyzed in Fig. 2 confirmed that the total culture yields were lower when cells were grown in bFGF/PDGF-AA or bFGF/PDGF-BB compared to CTL cultures.

### NS passage effects on OPCs yield from cultures

In order to evaluate if NS passage affects the final OPC yield, primary SVZ-derived cultures freshly prepared (Passage 0, P_0_) or NS cultures reamplified once (Passage 1, P_1_) or twice (Passage 2, P_2_) were treated with bFGF/EGF or with bFGF/PDGF-BB. The NG2^+^ and PDGFRα^+^ cell proportions were determined in each condition. As the NS passage increased, the overall immunopositve NG2^+^/PDGFRα^+^ cells increase in CTL treated cultures and decrease in the bFGF/PDGF-BB condition (S3 D Fig.). Although these experiments require future follow-up, we considered ending the culture treatments before their first passage to optimize OPC yield.

### OPC-enriched cultures generate mature OLs

**Fig 3:**
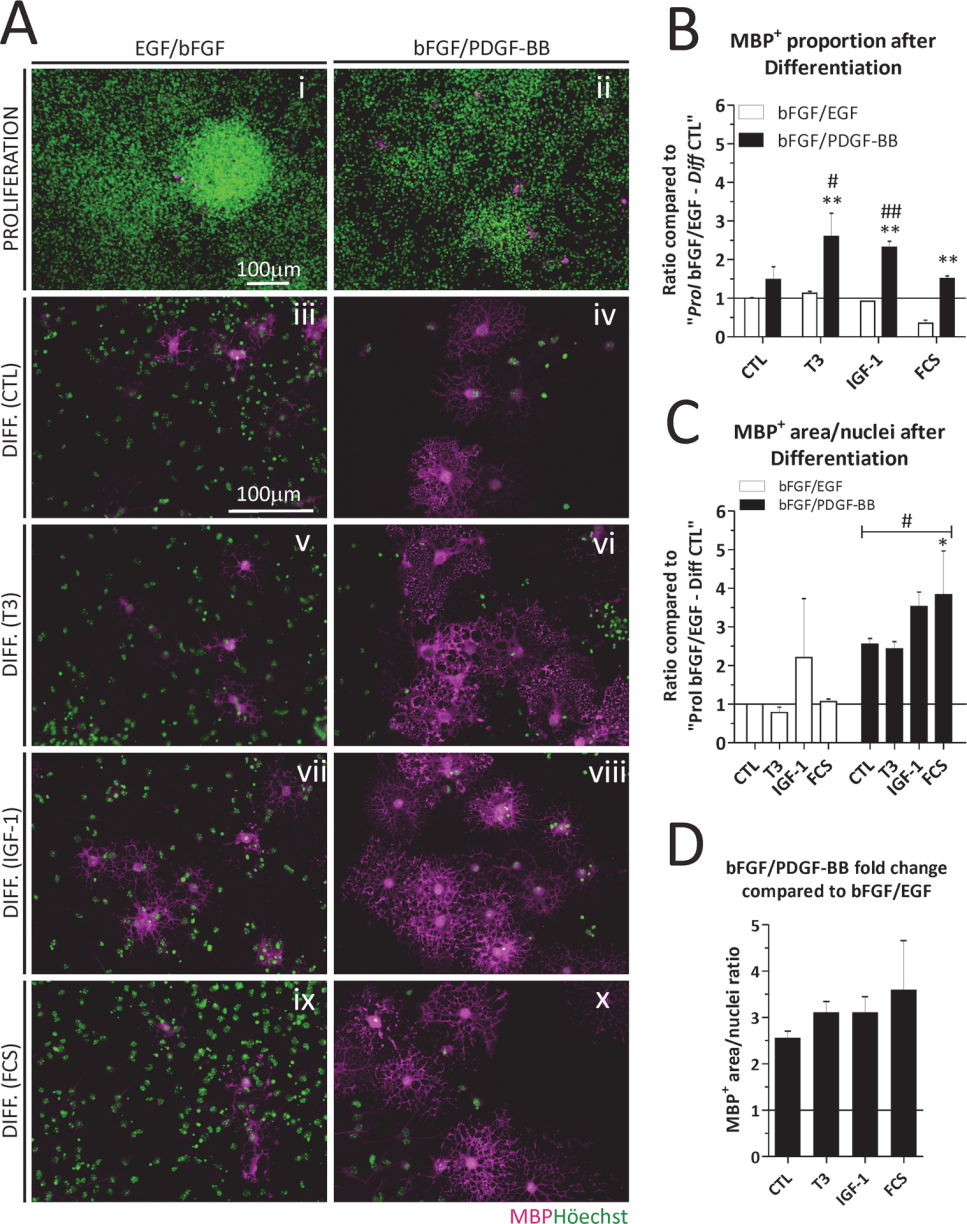
Oligodendrocyte maturation *in vitro* using WT-derived NS. A) Immunocytochemistry of NS cultures pre-treated during proliferation with bFGF/EGF or bFGF/PDGF-BB, and then exposed to differentiating conditions; No growth factors (CTL), Tri-iodothyronine (T3), Insulin-like Growth Factor 1 (IGF-1) and Fetal Calf Serum (FCS). B) Comparison of the proportion of MBP^+^ cells detected after differentiation between bFGF/EGF and bFGF/PDGF-BB pre-treated NS. C) Quantitation of the MBP^+^ area/nuclei ratio after differentiation in two independent cultures. Data in B and C was analyzed with a Two-way ANOVA and Bonferroni post-test to compare the effect of NS pre-treatment during proliferation (*) or the differentiation treatment compared to its CTL (#). D) Fold change of the MBP^+^ area/nuclei in bFGF/PDGF-BB compared to bFGF/EGF cultures. The bars in B, C and D represent the mean + SD. The scale bar in A (i) represents 100 μm for (i) and (ii), while the scale bar in A (iii) represents 100 μm for (iii) to (x). * = p < 0.05. ** = p < 0.01 and # = p < 0.05. DIFF.; Differentiation.

To confirm that bFGF/PDGF-BB pre-treated cultures from Act::EGFP mice were able to generate mature OL, we exposed them to differentiating media lacking growth factors. We also induce cell differentiation by addition of T3, IGF-1 or FCS as described in the S4 Fig. The presence of mature MBP^+^ OL was analyzed by ICC. During proliferation, cultures grown in bFGF/EGF or bFGF/PDGF-BB contained few MBP^+^ OL that had a poorly developed morphology; diameter and ramifications (Fig. 3Ai, ii). After differentiating bFGF/EGF pre-treated cultures (Fig. 3Aiii, v, vii and ix), there were no evident changes in mature OL proportions between the various differentiating conditions (white bars in Fig. 3B). However, there was a significant increase in MBP^+^ cell proportions after differentiating bFGF/PDGF-BB pre-treated cultures in the presence of T3, IGF-1 (Fig. 3Aiv, vi, viii and x, and black bars in Fig. 3B). The OL morphology is compared in Fig. 3C and D after differentiation. Adding T3, IGF-1 or FCS during differentiation had a significant impact on MBP^+^ cell proportions in bFGF/PDGF-BB pre-treated cultures compared to bFGF/EGF treated cultures. As a whole, we found a significant increase in the complexity of MBP^+^ OL cell morphology in bFGF/PDGF-BB pre-treated cultures compared to the bFGF/EGF pre-treatment (# in Fig. 3C). Nonetheless, FCS was the only differentiation treatment to significantly increase the cell morphology when analyzing the data with the Bonferroni post-test (* in Fig. 3C).

**Fig 4:**
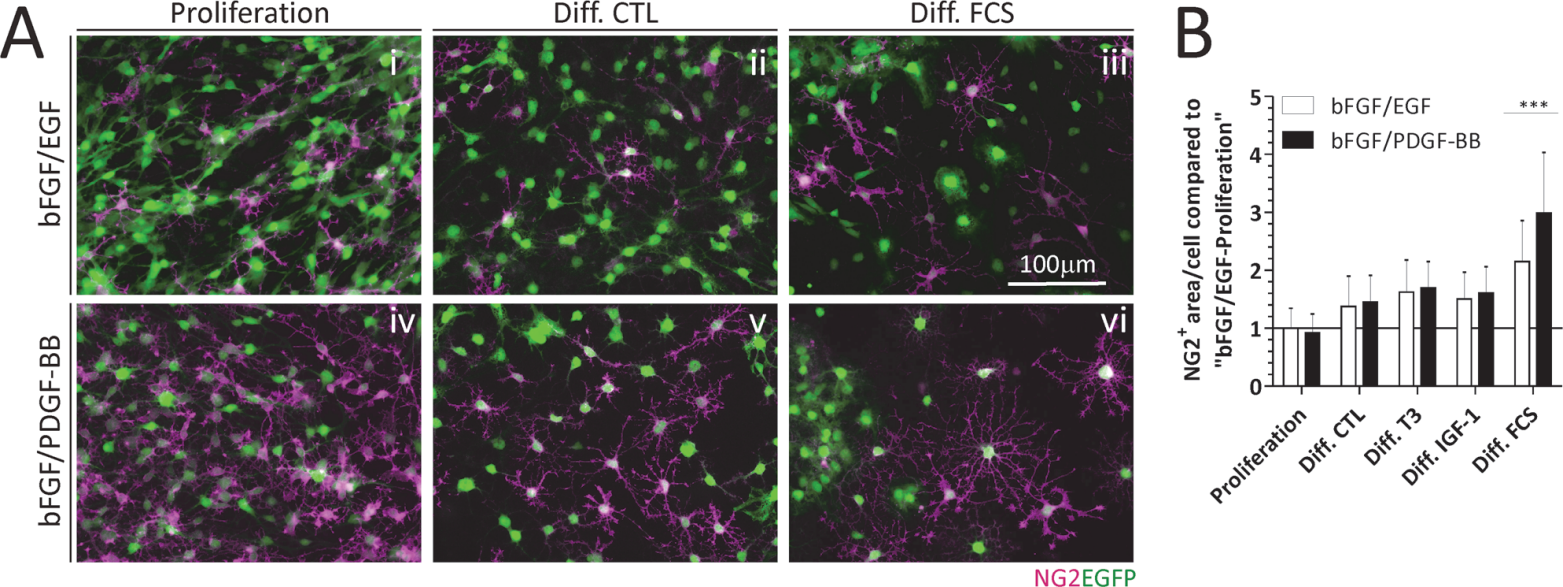
NG2 cells after differentiation in Act::EGFP-derived cultures. A) Representative ICC of NG2^+^ cells. B) The NG2^+^ pixel area per immunopositive cell is quantitated in each culture condition after differentiation. Quantitations were performed by analyzing at least 42 cells on a single cover slip for each treatment. The scale bar in A (iii) equals 100 μm for all images in A. Error bars represent the SD and *** = p < 0,001. Diff.; Differentiation.

After the differentiation stage in these Act::EGFP cultures, we noticed NG2^+^ cells were still present in cultures. There was a tendency to find more ramified NG2^+^ cells in differentiated cultures respect to proliferating ones (Fig. 4). The FCS-induced differentiation was the only condition that was able to capture a significant difference in the NG2^+^ cell morphology.

### Heparin as a strategy to improve cell culture yield

Since we were interested in improving the OPC yield in SVZ-derived NS cultures, we decided to analyze the effect of adding heparin to the cell cultures based on literature where heparin was proposed as a bFGF-stabilizer *in vitro*. Preliminary experiments were performed to evaluate the optimal Heparin concentration. Heparin at 1.25 U/L final concentration worked best for bFGF/PDGF-BB cultures (S5 A, B Fig.), while hampering the cell culture yield at higher concentrations.

**Fig 5:**
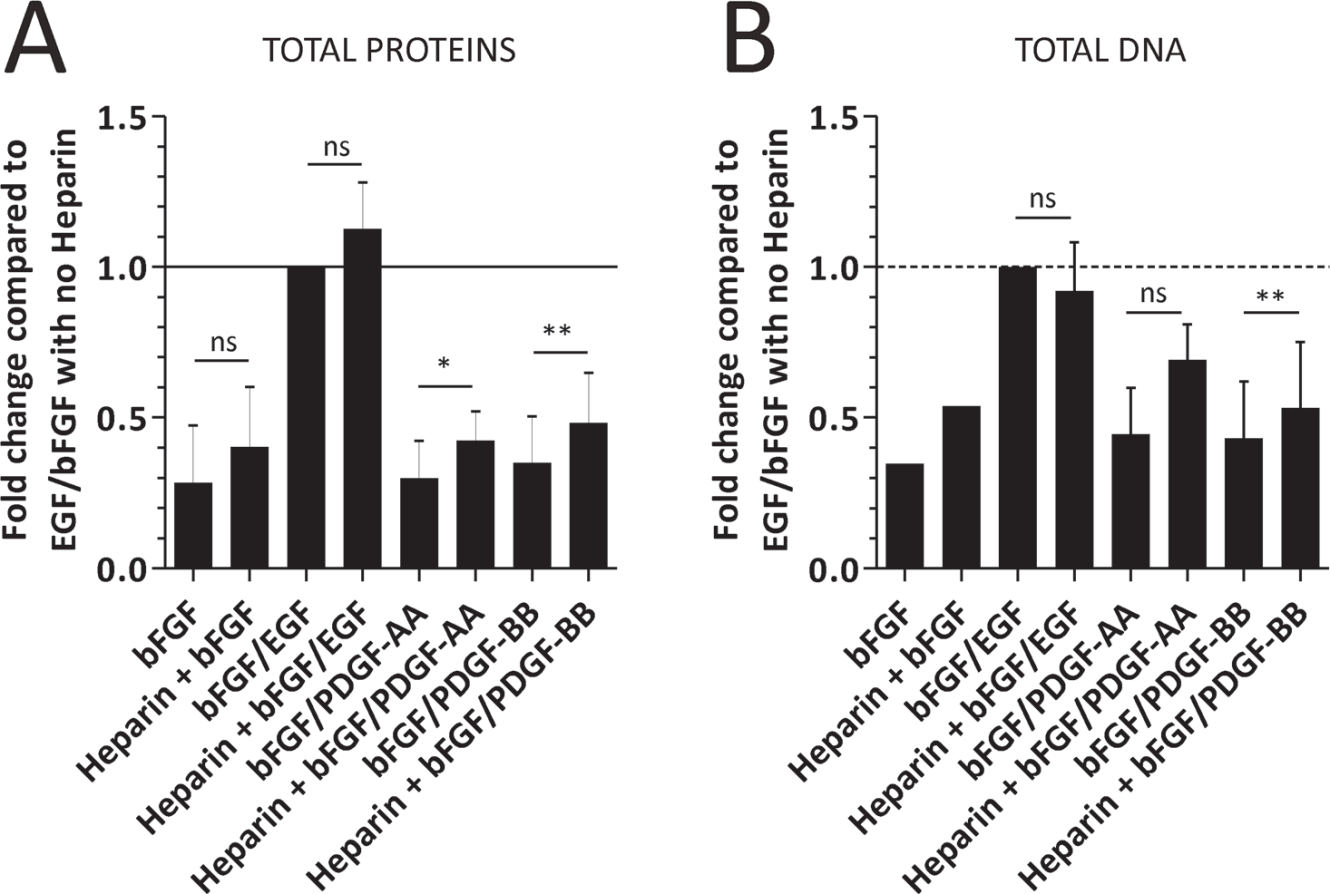
Heparin effects on cell culture yield from 3 WT and 2 CNP::EGFP mice. Total cell protein concentration (A) and DNA content (B) quantitation in NS obtained from cultures exposed to different growth factor combinations, and in the absence or presence of 1.25 U/L of Heparin in the culture media during proliferation. The error bars in both graphs represent the SD. Data was analyzed with a One-way ANOVA and Dunnet´s post-test, where the “bFGF/EGF” (without Heparin) treatment was established as the control.

The NS diameter length was compared between treatments in the absence and presence of 1.25 U/L Heparin, and among WT and Act::EGFP mice strains. There was a tendency to find larger NS in the bFGF/PDGF-BB-treated cultures in the presence of Heparin than in its absence (S5 C Fig.). The comparison of the different growth factor combinations on cell yield in the absence or presence of 1.25 U/L indicated that heparin effectively had an effect on bFGF (Fig. 5A, B and S5 D, E Fig.). Since we were most interested in increasing OPC yields, we analyzed Heparin effects on bFGF/PDGF-BB cultures. Heparin increases the bFGF/PDGF-BB treated culture yield by 40% or 20% when analyzing total proteins and DNA, respectively, and was found to be statistically significant. However, the cell culture yield in the bFGF/PDGF-BB conditions never reached comparable levels to those obtained with the CTL condition (bFGF/EGF).

### Oligodendrogenesis and oligodendrocyte maturation in the presence of Heparin

**Fig 6:**
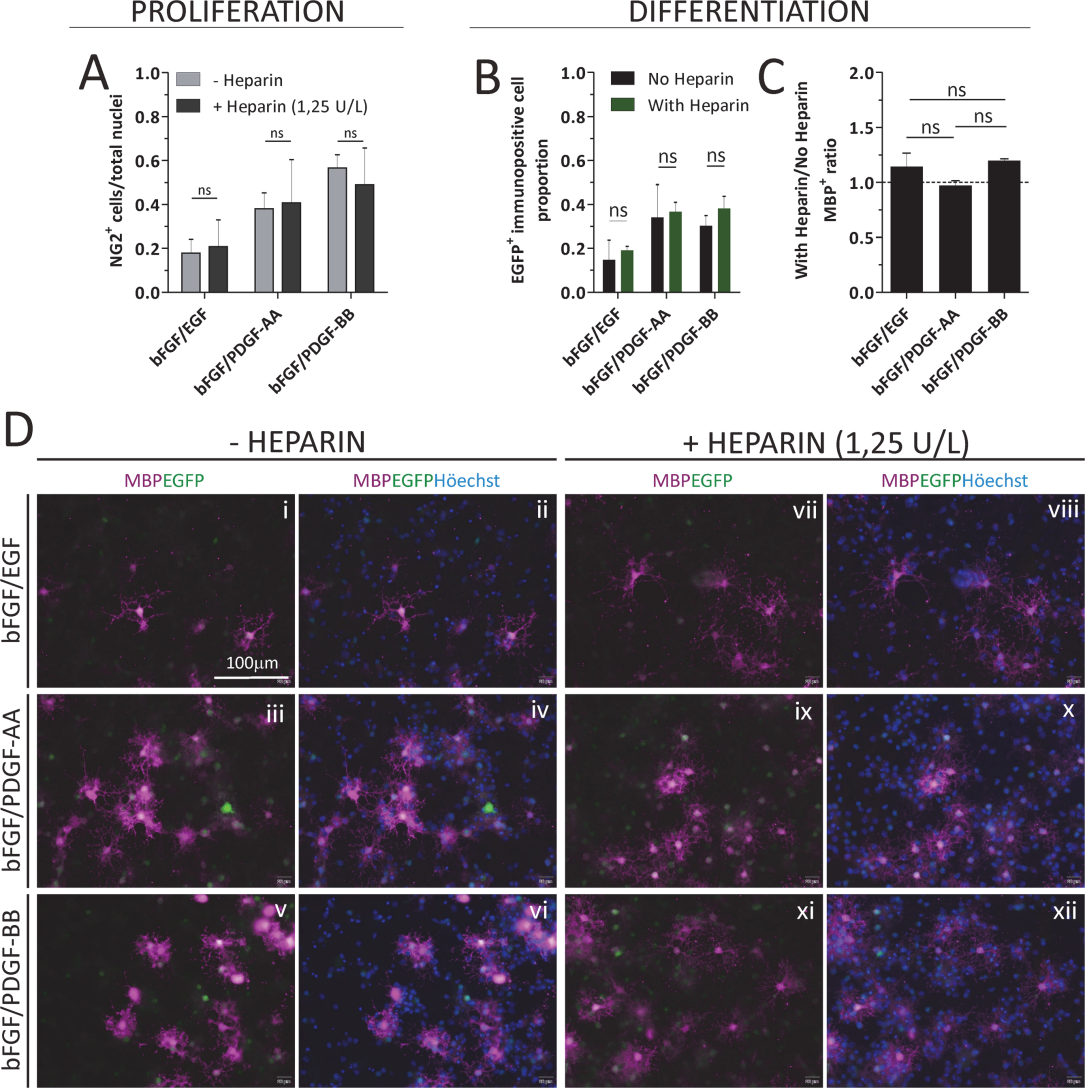
Heparin effects during oligodendrogenesis and oligodendrocyte maturation in CNP::EGFP mice cultures. A) The number of NG2^+^ cells at the end of the proliferation stage are expressed as a proportion respect to the total number of nuclei analyzed per treatment. More than 300 nuclei were analyzed for each treatment in 4 independent cultures. B, C) The proportion of GFP^+^ and MBP^+^ cells after differentiation is compared in Heparin (1.25 U/L) treated cultures against culture lacking Heparin. More than 600 nuclei were analyzed for a each treatment. Data analysis in graphs was performed using Student´s t Test. D) Representative images of CNP::EGFP mice derived cutlures demonstrating EGFP^+^ (green) and MBP^+^ (magenta) cells. Scale bar in D (i) represents 100 μm for all images in D. Each pair of columns in A (with or without Heparin) was analyzed with a Student´s t test. ns = not significant.

We used the CNP::EGFP mice to analyze the OPC maturation capacity in the presence or absence of Heparin. The proportion of NG2^+^ cells generated by PDGF-BB treatment was not significantly different when Heparin was used in the culture during proliferation at 1.25 U/L (Fig. 6A). These findings were evaluated in parallel by analyzing the EGFP expression in the same cultures of CNP::EGFP mice and we observed that adding Heparin to the cultures during proliferation did not alter the EGFP expression in either of the treatments (S6 Fig.). The proportions of EGFP and MBP expressing cells were used as indicators of OL commitment when using NS cultures derived from CNP::EGFP mice under standard differentiating conditions. The addition of Heparin 1.25 U/L to the culture media did not hamper the generation of cells belonging to the OL lineage when evaluating the EGFP^+^ or MBP^+^ cells by ICC (Fig. 6B, C). The OL generated during differentiation did not differ morphologically among Heparin treated or non-treated cultures (Fig. 6D).

### PDGF-BB pre-treated cultures generate OPC able to interact with neurons

**Fig 7:**
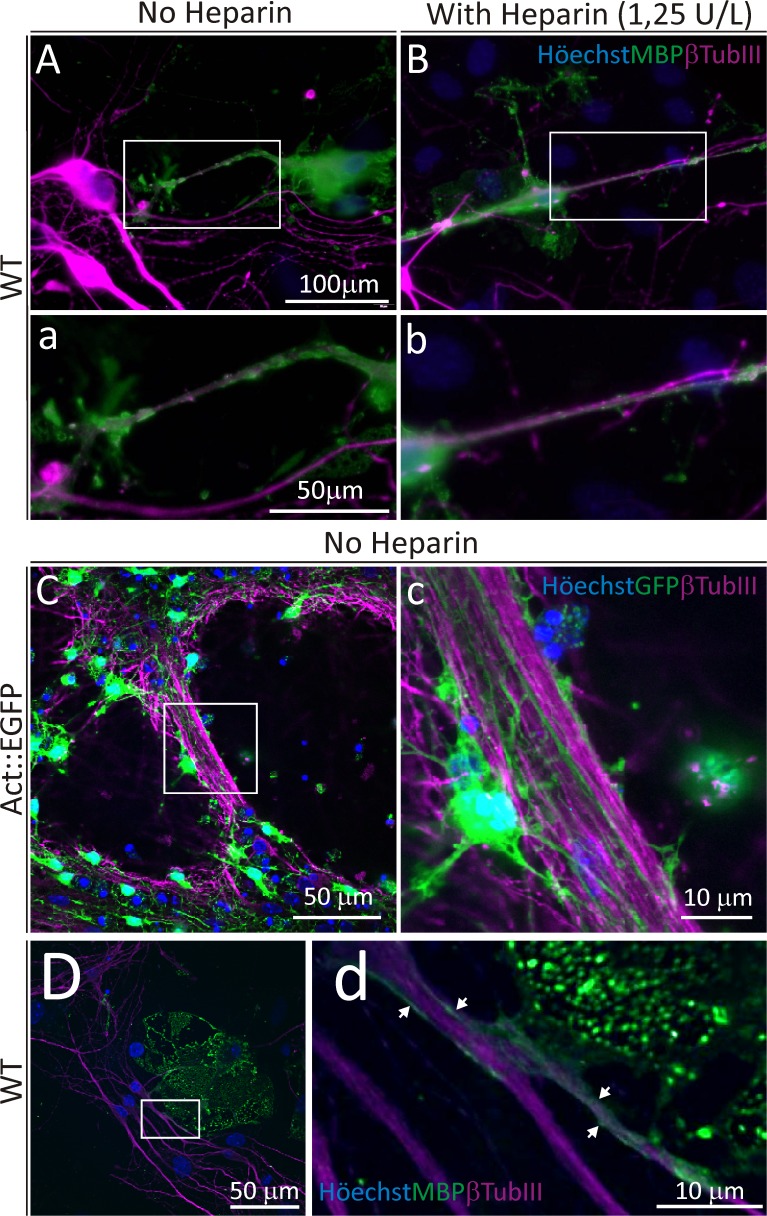
Confocal and epi-fluorescence microscopy images of WT neuron and Act::EGF NS co-cultures. Axonal fibers are labelled with βTubulin III (magenta) for all images. A, B) Epi-fluorescence images of MBP^+^ (green) and βTubIII cells belonging to bFGF/PDGF-BB cultures treated without (A) or with Heparin (B) during proliferation. The insets in *A* and *B* are shown in *a* and *b*, respectively. C, D) Confocal images of co-cultures of neurons and bFGF/PDGF-BB (with no added Heparin) pre-treated neurospheres. C) Image of EGFP^+^ cells from Act::EGFP mice and β-TubIII^+^ neurons. D) Confocal image of an MBP^+^ OL (from WT-derived neurospheres) interacting with a βTubIII^+^ neuron projection. The insets *C* and *D* is shown enlarged in *c* and *d*, respectively. Scale bar in A = 100 μm for A and B, scale bar in a = 50 μm for a and b.

A neuron-OPC *in vitro* co-culture model was used as a functional assay for OPC-enriched cultures. NS from WT mice were pre-treated with bFGF/PDGF-BB and then plated on fetal brain neurons from WT mice (Fig. 7). We made sure that no MBP^+^ OL were present in the neuron cultures before the plating the NS-derived OPC (data not shown). Cells were co-cultured for 6 days in DMEM/F12-B27 culture media. We detected the interaction of MBP^+^ cells with βTubulin III^+^ axonal projections, either in the absence or presence of Heparin using a conventional epi-fluorescence microscope (Fig. 7A, B). The OPC from Act::EGFP mice pretreated with bFGF/PDGF-BB were also plated onto WT-derived neurons (Fig. 7C). A 3D reconstruction of z-stack confocal images showed GFP expressing cells were able to accommodate their ramifications together with the neuronal axons (S7 Fig.).

In regions of lower axonal density, MBP^+^ cell membranes of WT mice-derived NS cultures we observed ensheathing βTubulin III^+^ ramifications (Fig. 7D). As a whole, these results indicated that OPC-enriched cultures produced by this method could effectively interact with neurons, and serve as a promising source of OPC for future *in vivo* or *in vitro* studies.

## DISCUSSION

The use of different PDGF isoform homodimers in the culture media allowed us to detect significant variations in the amount of NG2^+^/PDGFRα^+^ proportions when using murine SVZ-derived NS primary cultures of the C57BL6/j genetic background.

To analyze the culture system oligodendrogenic potential we chose the NG2 and PDGFRα markers to label OPC. We are aware of published literature describing PDGFRα expression on NPC [23, 48] and NG2 being detected in SVZ type C cells [49], glial progenitor cells [50] and neuron committed cells [51, 10]. Particularly, NG2^+^ cells have drawn attention and have been considered a fourth kind of non-neuronal cell type; the polydendrocytes [52, 53]. However, according to the larger body of literature and our previous findings, we considered the NG2^+^ and PDGFRα^+^ cells as committed to the earliest stages of OL development [54, 55].

Since the Olig2 transcription factor is not a OL-restricted biomarker and can be detected in astrocytes during development or SNC injury [56, 57, 58, 59], we purposely avoided studying OPC proportion variation in response to PDGF treatments by quantitating Olig2^+^ cells. Moreover, we found that more than half the GFAP^+^ cells in the CTL condition expressed Olig2. Since Olig2 and Sox2 greatly overlap [60], suggesting Olig2^+^ cells in SVZ-derived neurosphere cultures have a more immature phenotype than that of an OPC, we propose that the Olig2^+^/GFAP^+^ could be transient amplifying-like cells.

As a whole, both PDGF homodimers had differential impacts on the cell culture yield according their combinations with either EGF or bFGF. Noteworthy, while cell biomass yields were significantly lower when combining PDGF with bFGF than with EGF, the OPC proportions increased notoriously, being maximal with the bFGF/PDGF-BB combination. According to Grinspan et al. (1990) [61], PDGF isoforms have a proliferative effect on rat forebrain oligodendroglial cells. Particularly, the lower yields in bFGF/PDGF cultures compared to bFGF/EGF cultures is in agreement with Pedraza et al. (2008) [24] and Wang et al. (2013) [48]. The lower OPC proportions in EGF-treated cultures might be explained by the rapidly dividing NPC that express EGFR, similar to SVZ transient amplifying (Type C) cells [62] that could outweigh OPC proliferation rates. Additionally, EGFR^+^ cells that respond to the EGF ligand tend to give rise to less OPC than EGFR^-^ cells [63].

Since bFGF can participate on its own in driving oligodendrogenesis from progenitors [31], both bFGF and PDGF in this culture system would be increasing OPC proportion either by inducing OPC proliferation rate and/or by promoting NSC commitment to the oligodendroglial lineage. The more potent effects of PDGF-BB observed in our system compared to PDGF-AA is in agreement with data reported by Cohen et al. (1999) [64] that compare the effects of these PDGF isoforms on optic nerve progenitors.

Not only were we able to improve the overall OPC proportions in the NS culture system, but we were also able to demonstrate that after differentiation, the bFGF/PDGF-BB-exposed OL expressed MBP and developed into significantly more arborized cells than the EGF/bFGF-treated cells. The increase in the MBP^+^ cell morphology *in vitro* could be indicative of their *in vivo* myelinogenic capacity.

We also noticed NG2^+^ cells persisted even 6 days after growth factor removal from the culture media, similar to data described for spinal cord progenitor cultures by Kulbatski et al. (2007) [65]. This goes in line with the notion that NG2^+^ cells *in vivo* do not exit the cell cycle irreversibly, even during adulthood, unless they mature into myelinating OL [53]. In addition, we previously published that most BrdU^+^ cells persisting in differentiated rat NSC/NPC cultures were NG2^+^ [66]. However, adding FCS under differentiating conditions increased the NG2^+^ cell morphology and might have driven these cells to a post-mitotic pre-OL state.

An interesting point to discuss is the presence of a small fraction of cells in the NS culture systems that expressed only one of the OPC markers but conserved a typical OPC morphology. This is in agreement with *in vivo* and *in vitro* data published by He et al. (2009) [67] and Silvestroff et al. (2013) [66], respectively. Since all NG2^+^ cells had a ramified OPC morphology, we ruled out they could be NG2^+^ pericytes or vascular cells. Besides the possible chronological differences in activation and silencing of both OPC markers (NG2 and PDGFRα), we question whether OPCs necessarily need to go through an NG2^+^/PDGFRα^+^ stage during maturation. As a whole, these results contribute in setting a far more complex panorama than originally expected in terms of the NG2 restriction to the OL lineage.

In regards to methodology, the NS radius size measurement is an indirect parameter that reflects cell proliferation as a whole within each NS [68]. Although quimerism has been documented in NSC cultures under the form of NS, even at low seeding densities [69, 70], we were still able to notice significant correlation between the average NS size and the total cell protein and DNA quantitative values, and overall EGFP fluorescence. Moreover, our interest was set to analyze the NS culture yield rather than the NS clonality. If one does not have access to appropriate equipment to automate the NS size quantitation, such as a large particle cytometer [71], manually measuring the size of hundreds of NS becomes a time-consuming approach. Quantitating total NS proteins, DNA or the expression of constitutive reporter genes are alternate methods for analyzing the overall NSC/NPC primary culture yield, since they can be used to measure larger replicate numbers, in a feasible and high throughput fashion.

Based on previous literature [72], we used Heparin to improve cell biomass yields in the bFGF/PDGF-BB cultures. Although Heparin had a positive effect on the overall biomass yield at 1.25 U/L, it had deleterious effects at higher concentrations. Addition of Heparin during NS formation produced higher numbers of NG2^+^ cells and after differentiation generated more MBP^+^ mature OL. With this culture protocol we were able to lower contaminating PDGFRα^-^/NG2^-^ cells to a minimum of 25% of total cells in the best conditions before the first passage, so further increasing OPC proportions will continue to be the aim of our future research.

The co-culture system allowed us to obtain detailed information regarding intercellular interactions between OPC enriched EGFP^+^ cell cultures and βTubulin III^+^ neurons. The bFGF/PDGF-BB/Heparin-treated cells were able to extend lengthy membrane arborizations along neuronal cell projections in a cell context that intended to emulate an *in vivo* context.

Overall, the data provided in this work demonstrates that the OPC yield from SVZ-derived cell cultures can be improved with the PDGF-BB isoform in comparison to classical bFGF-EGF, or PDGF-AA-based protocols. Additionally, it would be expected that the OPC-enriched cultures obtained from NSC/NPC exposure to PDGF-BB and heparin generate cells suitable for cell transplantation for treating demyelinating diseases.

## Supporting Information

S1 Fig A-D) PDGFRα^+^ cells and EGFP^+^ are shown in Act::EGFP (A, B) and CNP::EGFP (C, D) derived cultures after bFGF/EGF and bFGF/PDGF-BB-treated cultures. E, F) NG2^+^ and EGFP^+^ cells present in CNP::EGFP-derived NS cultures. G) The GFAP^+^ and βTubIII^+^ cells were analyzed in WT NS cultures after 6 days in the presence of differente growth factor combinations. Data belongs to two independent cultures where more than 500 nuclei were analyzed per condition. H-O) Representative images of Olig2^+^ nuclei in CNP::EGFP-derived cultures under bFGF/EGF or bFGF/PDGF-BB-treated cultures. Scale bar in A = 100 μl forimages A-F and H-O.
(TIF)

S2 Fig GFAP and Olig2 immunodetection in WT-derived cultures. A-D) Representative images of cells expressing GFAP^+^ and/or Olig2^+^ cells in bFGF/EGF or bFGF/PDGF-BB-treated cultures, where GFAP^+^/Olig2^+^ double labelled cells are indicated with a yellow arrowhead. The inset in *B* is shown enlarged in *b*. E) Quantitative analysis of immonopositive cell proportions. The GFAP^+^/Olig2^+^ proportions were compared among treatments with Student´s t test. ** = p < 0.01. Scale bar in A = 100 μm in A-D.
(TIF)

S3 Fig Neurosphere size analysis and comparison between WT and Act:::EGFP mice strains. A) The NS size is analyzed according to the NS radius length. Data is expressed as a percentage of the EGF/bFGF-treated cultures. White bars belong to WT cultures and black bars belong to Act::EGFP-derived cultures. Each bar represents the mean value of cultures belonging to 6 different mice. Bars for each strain were compared with a One-Way ANOVA and Dunnett´s post-test, where EGF/bFGF bars were established as controls. B) Representative bright field images of WT NS in culture after treatment with different growth factor combinations. C) Bright field and fluorescent microscopy images of plated NS under EGF/bFGF treatment belonging to WT and Actin::EGFP mice. The exposure times for EGFP fluorescence images are expressed in milliseconds (ms). D) Quantitation of NG2^+^ and/or PDGFRα^+^ cells is shown for cell cultures at passages 0, 1 and 2 (P_0_, P_1_ or P_2_, respectively) treated with different growth factor combinations. At least 500 nuclei were analyzed in each condition. The scale bar in B (i) equals 200 μm for all images in B. The scale bar in C (ii) equals 100 μm for all images in C. Error bars in A and D represent the SD. * = p < 0.05, ** = p < 0.01, *** = p < 0.001.
(TIF)

S4 Fig Schematic representation of the time course of the experimental procedures used to amplify and differentiate the SVZ-derived cultures. Cells in either CTL (bFGF/EGF) or bFGF/PDGF-BB treated cultures were amplyfied as suspended NS for 6 days. On the 6^th^ day, NS were mechanically dissociated and plated on poly-L-Lysine coated cover-slips for 2 aditional days under the same proliferative treatment. On the 8^th^ day in vitro, the culture media was changed to one of the varios differentiation conditions for an aditional 6 days, after which the treatment ended by fixing the cells before the immunocytochemistry protocol.
(TIF)

S5 Fig Total proteins concentration and DNA content in WT NS cultures exposed to different growth factor combinations during proliferation. Data in A and D correspond to total protein quantitations and data in B and E belong to total DNA quantitations. A, B) Protein and DNA content in bFGF/EGF, or bFGF/PDGF-BB, cultures exposed to different Heparin concentrations. Data belongs to at least two independent cultures for each condition and was analyzed with a Two-Way ANOVA and Bonferroni post-test. The asterisks indicate if different Heparin concentrations significantly affect protein or DNA content compared to cultures lacking Heparin supplementation. C) The NS diameter (μm) is compared under different culture conditions in the presence or absence of Heparin (1.25 U/L) and among WT and Act::EGFP mice. D, E) Protein and DNA fold change for different culture conditions when 1.25 U/L of Heparin are added compared to matched culture conditions lacking Heparin. Error bars represent the SD, where * = p < 0,05 and ** = p < 0,01.
(TIF)

S6 Fig Fluorometric quantitation of the EGFP fluorescence in 3 independent cultures from CNP::EGFP mice. The SVZ derived NS cultures were generated under treatment with different growth factor combinations. After 6 days *in vitro*, we fluorometrically analyzed the DNA content and EGFP expression. The EGFP fluorescence was normalized to the amount of DNA (EGFP/Höechst ratio) in each condition, and then was compared between cultures containing or lacking Heparin (1.25 U/L). Error bars represent the SD.
(TIF)

S7 Fig Three dimensional reconstruction from a confocal microscope z-stack file consisting of 10 overlapped planes 1 μm apart. EGFP is shown in green and βTubulin III in red. The scale bar represents 20 μm.
(TIF)
