# Supplementary figures and images for: Optimizing Culture Medium Composition to Improve Oligodendrocyte Progenitor Cell Yields In Vitro from Subventricular Zone-Derived Neural Progenitor Cell Neurospheres
Source: PLoS One. 2015 Apr 2;10(4):e0121774. doi: 10.1371/journal.pone.0121774 (PMC4383518; doi:10.1371/journal.pone.0121774)

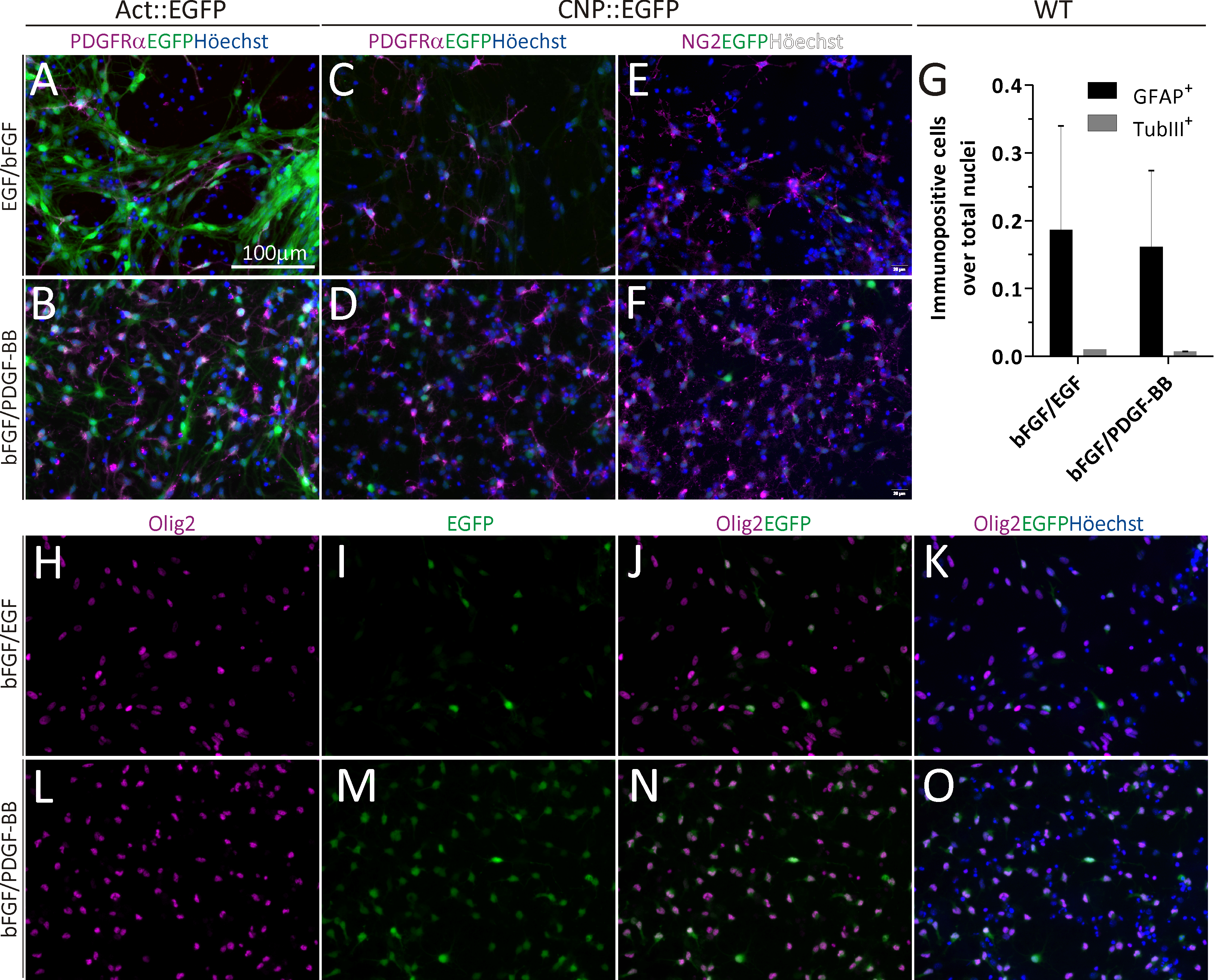

Supplement: S1 Fig — E, F) NG2+ and EGFP+ cells present in CNP::EGFP-derived NS cultures. G) The GFAP+ and βTubIII+ cells were analyzed in WT NS cultures after 6 days in the presence of differente growth factor combinations. Data belongs to two independent cultures where more than 500 nuclei were analyzed per condition. H-O) Representative images of Olig2+ nuclei in CNP::EGFP-derived cultures under bFGF/EGF or bFGF/PDGF-BB-treated cultures. Scale bar in A = 100 μl forimages A-F and H-O. (TIF) [file pone.0121774.s001.tif]

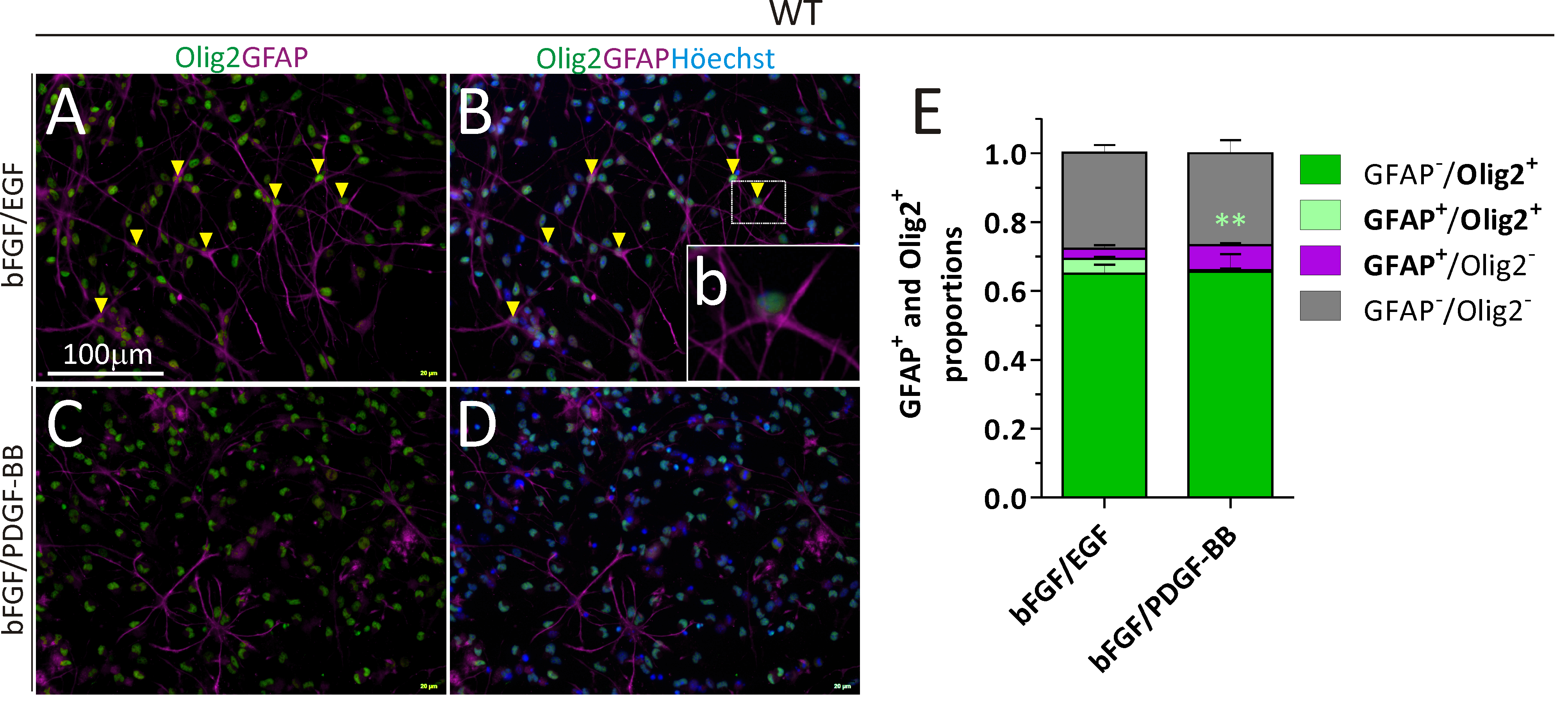

Supplement: S2 Fig — A-D) Representative images of cells expressing GFAP+ and/or Olig2+ cells in bFGF/EGF or bFGF/PDGF-BB-treated cultures, where GFAP+/Olig2+ double labelled cells are indicated with a yellow arrowhead. The inset in B is shown enlarged in b. E) Quantitative analysis of immonopositive cell proportions. The GFAP+/Olig2+ proportions were compared among treatments with Student´s t test. ** = p < 0.01. Scale bar in A = 100 μm in A-D. (TIF) [file pone.0121774.s002.tif]

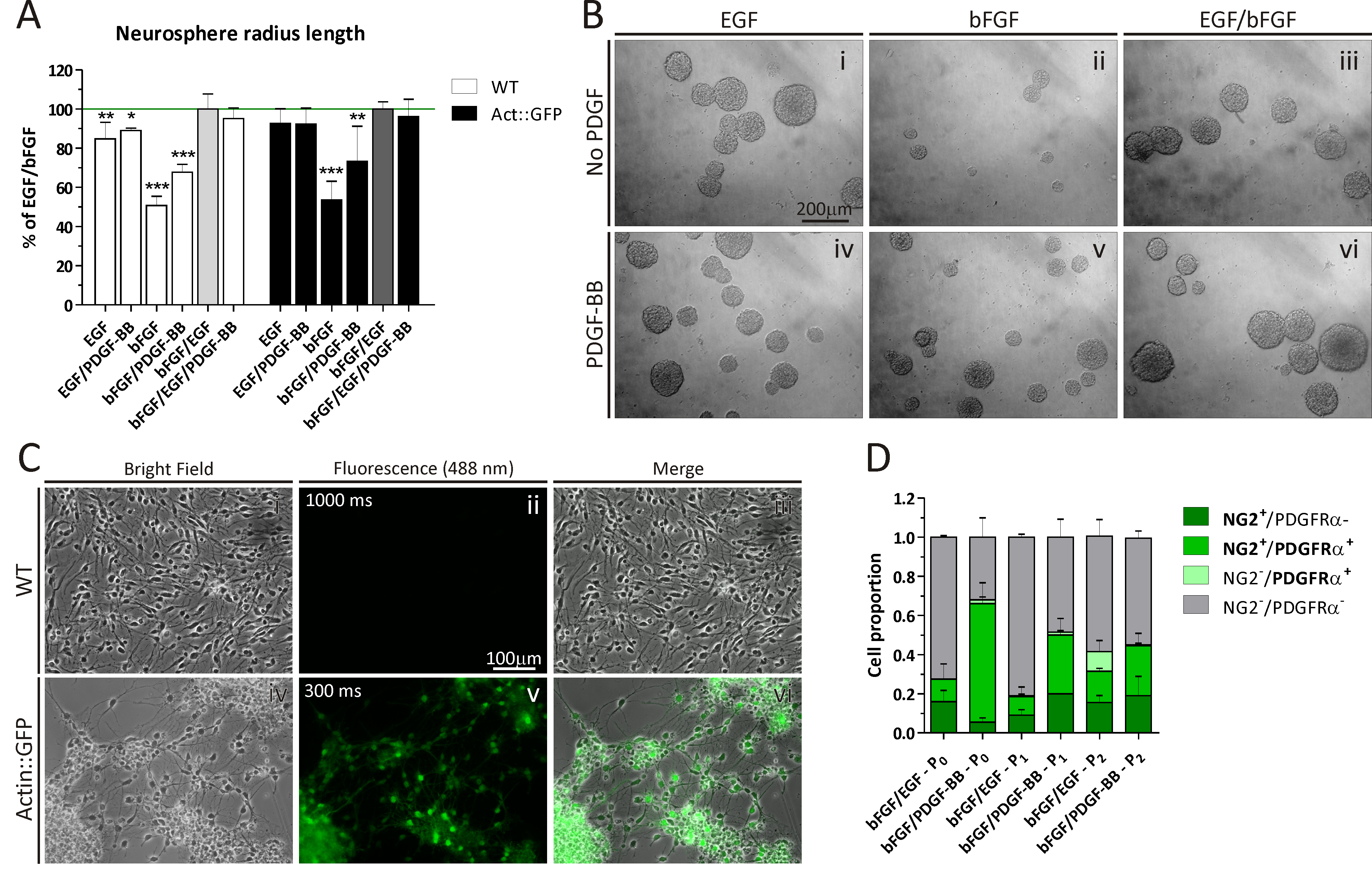

Supplement: S3 Fig — A) The NS size is analyzed according to the NS radius length. Data is expressed as a percentage of the EGF/bFGF-treated cultures. White bars belong to WT cultures and black bars belong to Act::EGFP-derived cultures. Each bar represents the mean value of cultures belonging to 6 different mice. Bars for each strain were compared with a One-Way ANOVA and Dunnett´s post-test, where EGF/bFGF bars were established as controls. B) Representative bright field images of WT NS in culture after treatment with different growth factor combinations. C) Bright field and fluorescent microscopy images of plated NS under EGF/bFGF treatment belonging to WT and Actin::EGFP mice. The exposure times for EGFP fluorescence images are expressed in milliseconds (ms). D) Quantitation of NG2+ and/or PDGFRα+ cells is shown for cell cultures at passages 0, 1 and 2 (P0, P1 or P2, respectively) treated with different growth factor combinations. At least 500 nuclei were analyzed in each condition. The scale bar in B (i) equals 200 μm for all images in B. The scale bar in C (ii) equals 100 μm for all images in C. Error bars in A and D represent the SD. * = p < 0.05, ** = p < 0.01, *** = p < 0.001. (TIF) [file pone.0121774.s003.tif]

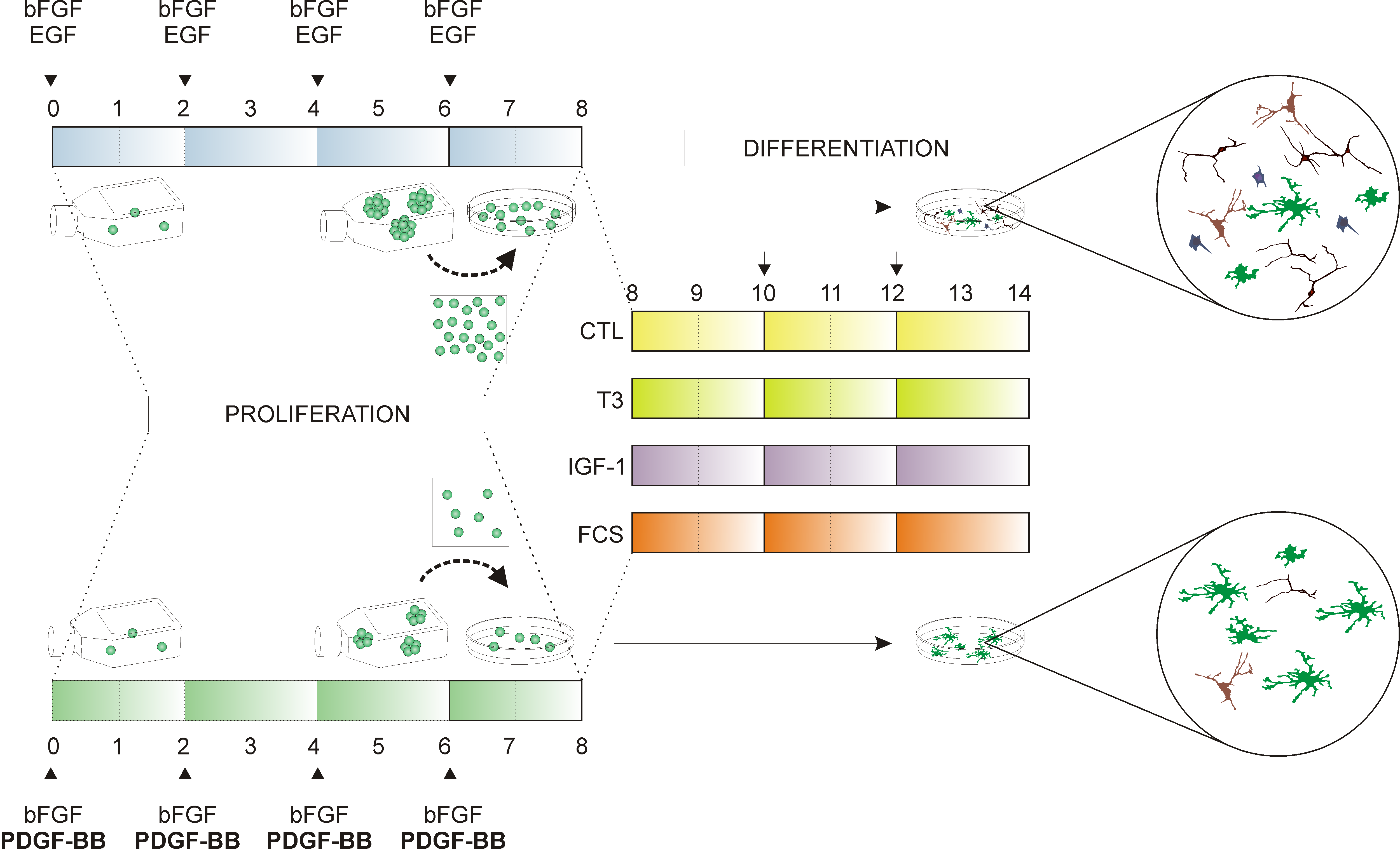

Supplement: S4 Fig — Cells in either CTL (bFGF/EGF) or bFGF/PDGF-BB treated cultures were amplyfied as suspended NS for 6 days. On the 6th day, NS were mechanically dissociated and plated on poly-L-Lysine coated cover-slips for 2 aditional days under the same proliferative treatment. On the 8th day in vitro, the culture media was changed to one of the varios differentiation conditions for an aditional 6 days, after which the treatment ended by fixing the cells before the immunocytochemistry protocol. (TIF) [file pone.0121774.s004.tif]

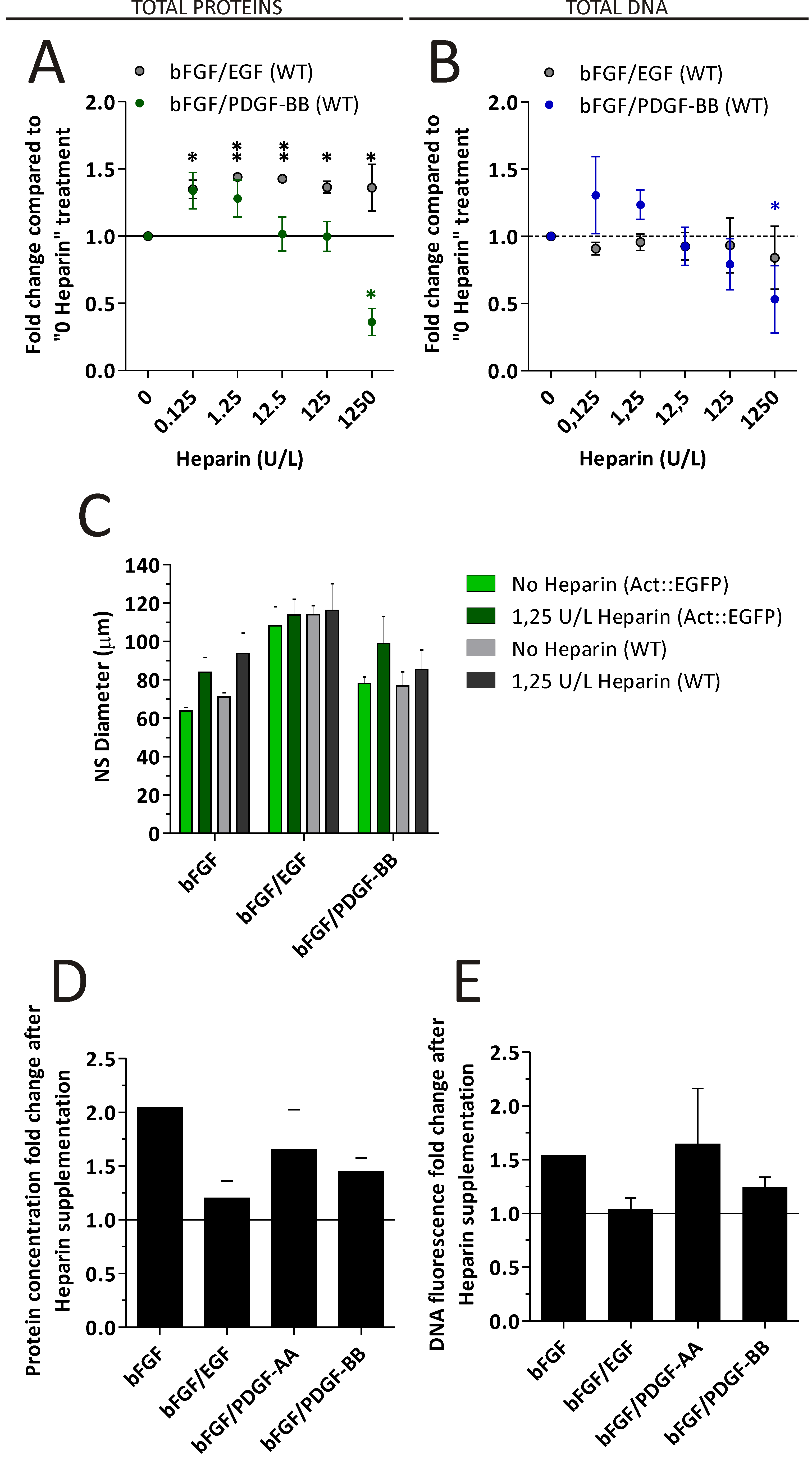

Supplement: S5 Fig — Data in A and D correspond to total protein quantitations and data in B and E belong to total DNA quantitations. A, B) Protein and DNA content in bFGF/EGF, or bFGF/PDGF-BB, cultures exposed to different Heparin concentrations. Data belongs to at least two independent cultures for each condition and was analyzed with a Two-Way ANOVA and Bonferroni post-test. The asterisks indicate if different Heparin concentrations significantly affect protein or DNA content compared to cultures lacking Heparin supplementation. C) The NS diameter (μm) is compared under different culture conditions in the presence or absence of Heparin (1.25 U/L) and among WT and Act::EGFP mice. D, E) Protein and DNA fold change for different culture conditions when 1.25 U/L of Heparin are added compared to matched culture conditions lacking Heparin. Error bars represent the SD, where * = p < 0,05 and ** = p < 0,01. (TIF) [file pone.0121774.s005.tif]

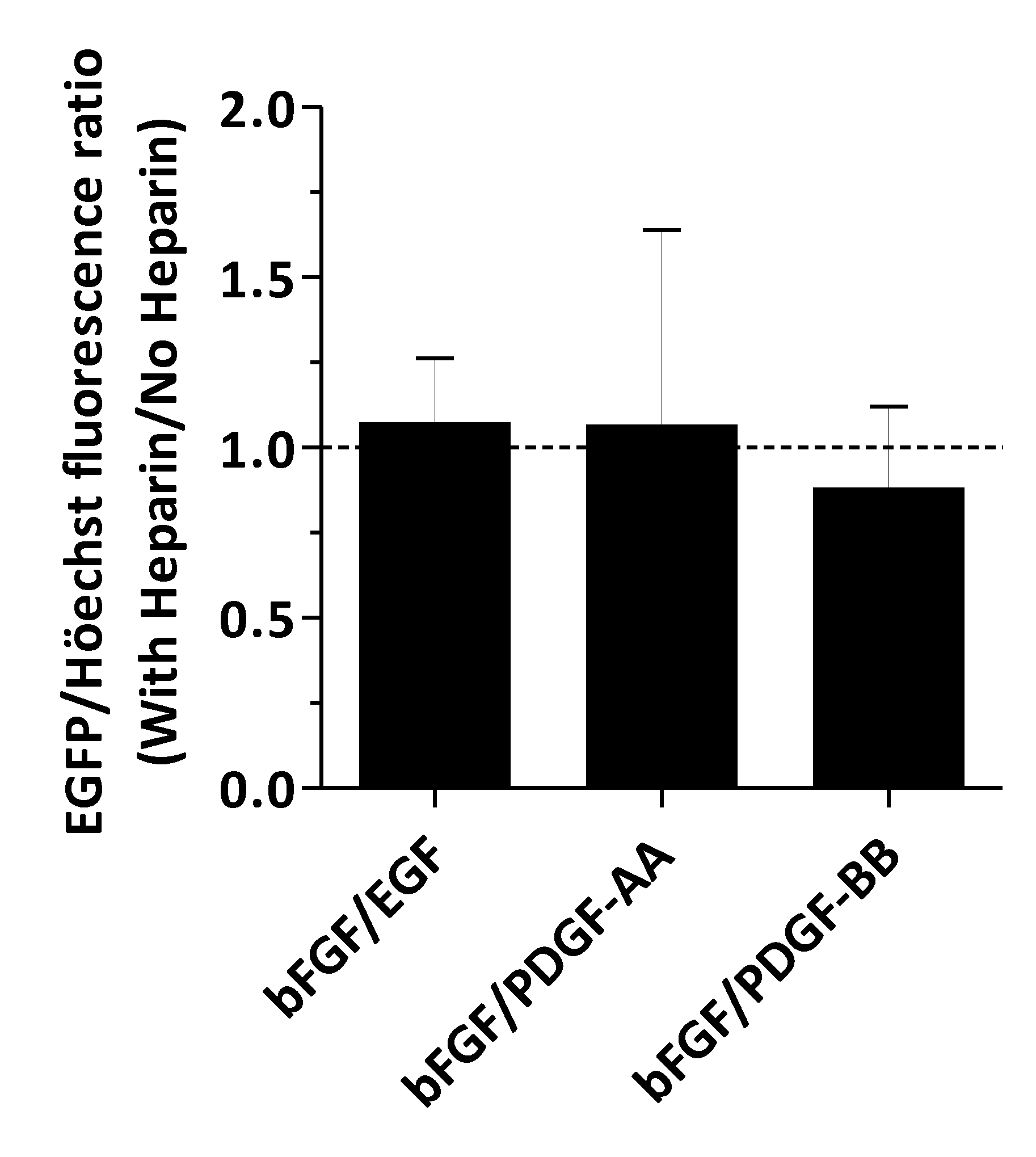

Supplement: S6 Fig — The SVZ derived NS cultures were generated under treatment with different growth factor combinations. After 6 days in vitro, we fluorometrically analyzed the DNA content and EGFP expression. The EGFP fluorescence was normalized to the amount of DNA (EGFP/Höechst ratio) in each condition, and then was compared between cultures containing or lacking Heparin (1.25 U/L). Error bars represent the SD. (TIF) [file pone.0121774.s006.tif]

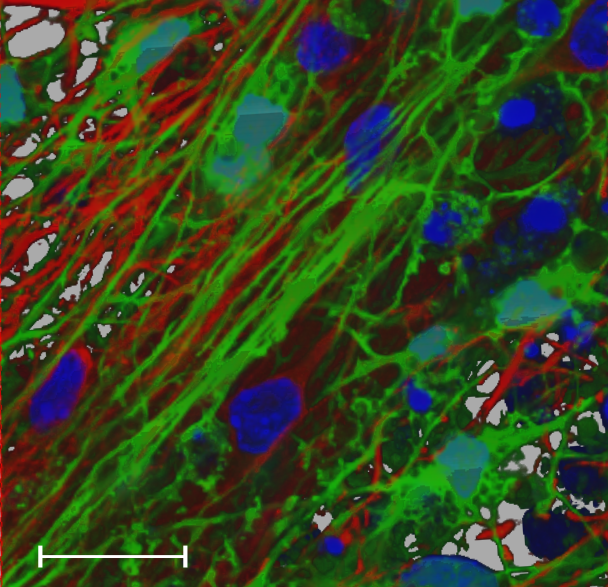

Supplement: S7 Fig — EGFP is shown in green and βTubulin III in red. The scale bar represents 20 μm. (TIF) [file pone.0121774.s007.tif]
